# Reporting Discrepancy Resolved by Findings and Time in 2947 Emergency Department Ankle X-rays

**DOI:** 10.1007/s00256-019-03317-7

**Published:** 2019-11-21

**Authors:** Thomas James York, P. J. Jenkins, A. J. Ireland

**Affiliations:** 1 St Mary’s Hospital, Praed Street, London, W2 1NY UK; 2 Department of Orthopaedic Surgery, Glasgow Royal Infirmary, 84 Castle Street, Glasgow, G4 0SF UK

**Keywords:** Ankle fractures, X-ray reporting, Reporting discrepancy, Time resolved discrepancy, Reporting errors

## Abstract

**Aims:**

To identify common errors in ankle X-ray reporting between initial interpretation and final assessment at the virtual fracture clinic. Also, to assess time of initial reporting as a causative factor for discrepancy.

**Methods:**

Two thousand nine hundred forty-seven final reports were reviewed by standard of agreement to the initial interpretation. Where discrepancy was found, it was classified and collated by specific finding. Comparison was made between reports with discrepancy and the complete dataset, allowing rates of error by finding to be established. The reports containing discrepancy were further classified by time period, this was compared against an expected value to establish if initial reporting outside of routine working hours was as accurate as that conducted within routine working hours.

**Results:**

94.4% of reports were in agreement with the initial interpretation, 2.9% contained minor discrepancy, and 2.7% major discrepancy. In 45.6% of reports there was no radiologically observable injury. 16.4% of reports contained a lateral malleolar fracture, most commonly Weber type B. 40.0% of all navicular fractures, and 33.3% of all cuboidal fractures were not commented upon in the initial reporting. Lower rates of more frequently observed findings were missed with 2.5% of Weber type B fractures not commented upon. An increased proportion of major discrepancy reports were generated from 00:00 to 07:59 (expected = 15.0%, observed = 22.2%; *p = 0.07908*). Similarly, a greater than expected number of minor discrepancy reports were found between 20:00 and 23:59 (expected = 18.0%, observed = 34.1%, *p = 0.00025*).

**Conclusions:**

The initial reporting of ankle X-rays in the emergency department is performed to a high standard, however serious missed findings emphasise the need for timely senior review. Reporters should increase their awareness of navicular, cuboid, talar, and Weber A fractures which were missed at disproportionate rates. This study also finds evidence to support increased rates of error in initial reporting of ankle X-rays outside of normal working hours (17:00–07:59), particularly with a significantly increased rate of minor discrepancy seen from 20:00 to 23:59.

## Introduction

Ankle injuries represent a significant proportion of emergency department work. They are the most common musculoskeletal injury [1] and of those presenting to the emergency department with ankle injury as their primary complaint, 15% will have an ankle fracture [2].

Recent research has indicated the annual incidence of ankle fractures is as high as 169 per 100,000 in Northern European populations [3]. In the elderly patient, they are amongst the 3 most common fracture types, along with fractures of the hip and distal radius [4].

Whilst the widespread adoption of the Ottawa Ankle Criteria has reduced unnecessary X-ray imaging of the ankle [5], they still set a relatively low threshold for investigation [6] and a busy emergency department is likely to conduct many hundreds of these per year.

The high rate of requesting and perceived ordinariness of these injuries means it frequently falls to more junior physicians to make the first assessment of an ankle radiograph. Despite this, inadequate treatment of radiologically identifiable injuries results in significant complication; unstable syndesmosis, cartilage degeneration, and mal-union may all lead to loss of function and pain [7]. This makes a timely, accurate assessment critical to a patient’s initial management.

In the associated department at Glasgow Royal Infirmary, the initial emergency department interpretation of an ankle X-ray is reviewed at a virtual fracture clinic where a definitive report is issued. This study sought to investigate cases where there was discrepancy between these two assessments. The primary objective being to identify commonly missed radiological findings on the initial assessment of an ankle X-ray.

The secondary objective involved a working-time resolved analysis of discrepancy, the aim being to establish the time of initial interpretation as a potential factor in the likelihood of reporting disagreement. Such a link, between clinical errors and non-standard working hours, has been increasingly reported in medical literature [8, 9] but is less tested in this specific domain.

## Patients and methods

This study takes the form of a retrospective audit performed at a University Teaching Hospital, serving a metropolitan area of approximately 350,000 people. The radiology department administrative database was examined to produce an anonymised list of 6886 sequential ankle X-rays, comprising a standardised anteroposterior and lateral view. These were taken between October 2011 and January 2014.

For each of these an initial report of findings was issued by the requesting physician during the emergency department visit. Doctors generating this report ranged in experience from their second year of practice to senior registrars and consultants.

All ankle X-rays were then reviewed by a consultant MSK radiologist and consultant orthopaedic surgeon at a virtual fracture clinic. This generated a final report of findings, including an assessment of agreement with the initial report to which they had access.

This was categorised as ‘agreement’ where no difference of interpretation was found, ‘minor disagreement’ where there was discrepancy not judged to require change to the patient’s management, and ‘major disagreement’ where intervention was required.

The reports were then screened to exclude those where an assessment of agreement was not recorded or was insufficiently recorded. Following this, a total of 2947 suitable reports remained for inclusion. A single observer reviewed these for standardised written descriptors to identify the frequency of radiological findings.

The frequency of each finding was collated across the data series as a whole and, in those reports where discrepancy was found, compared between the initial and final report.

To address the study’s secondary aim, the 2947 total reports and 166 discrepancy reports were further categorised by time period during which the initial report was generated: 08:00–16:59, 17:00–19:59, 20:00–23:59, and 00:00–07:59. These intervals reflect shift patterns in the emergency department and extended duration shifts.

An expected number of discrepancy reports for each time period was generated on the basis of a null hypothesis. This stipulated that the number of reports containing discrepancy would be directly proportional to the number of initial reports generated, therefore that time period had no impact on reporting accuracy. Comparison was then made between the expected and observed values to test if this was the case.

Biostatistical analysis was performed using Chi square testing for goodness-of-fit to assess the significance of variation in reporting errors. The conventional threshold of *p value < 0.05* was set as the cut off for significance.

### Ethical and research approval

The methodology of this study and the database used therein was approved by radiology department data release, and local Caldicott and audit committees. No research ethics committee opinion was required as treatment was not allocated or concealed, and patients were not contacted.

## Results

### Overall findings

The most common finding across all included reports was of no radiologically observable injury, seen in 1345 reports (45.6%). There were a total of 484 reports (16.4%) demonstrating a fracture of the lateral malleolus; 47 (1.6%) categorised as Weber type A, 315 (10.7%) type B, and 122 (4.1%) type C. Taken in combination, these injuries were the most common bony finding.

There were 215 reports (7.3%) including fracture of the medial malleolus, and 75 (2.5%) with a posterior malleolus fracture. Polymalleolar fractures were found less frequently than unimalleolar, bimalleolar fractures appeared in 96 (3.3%) and trimalleolar in 49 (1.7%).

Ankle mortice dislocation was seen in 104 reports (3.5%). There were a total of 67 (2.3%) including a fracture of the hindfoot; 35 (1.2%) calcaneal fractures and 32 (1.1%) talar fractures.

15 reports (0.5%) showed navicular fracture and a smaller number, 6 (0.2%) showed cuboid fracture. There were 19 (0.6%) with a fracture to the base of the 5th metatarsal.

The least commonly reported finding was of suspected osteomyelitis, recorded in 3 reports (0.1%) See Table 1.

**Table 1:**
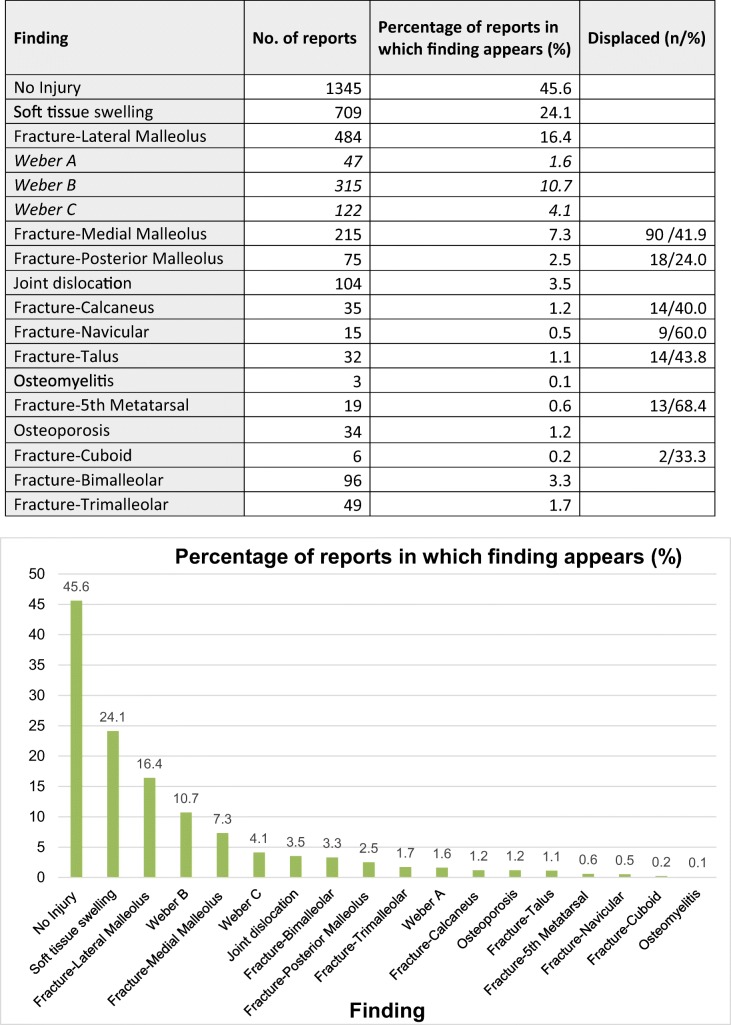
Total incidence

Total incidence of findings across the included 2947 reports

### Reporting discrepancy

In 2781 reports (94.4%) there was agreement with the initial findings. There were 85 (2.9%) that recorded minor discrepancy, and 81 (2.7%) with major discrepancy.

#### Minor discrepancy

In those 85 reports with minor discrepancy there were 48 (56.5%) with no radiologically observable injury, representing a false positive in the initial findings.

The remaining 37 reports (43.5%) included a missed finding; a false negative. In 24 (28.2%) soft tissue swelling had not been initially commented upon. There were 6 (7.1%) missed fractures of the lateral malleolus, all characterised as Weber A. A further 4 (4.7%) medial malleolar, and 3 (3.5%) posterior malleolar fractures were found not to have been highlighted in the initial report See Table 2.

**Table 2:**
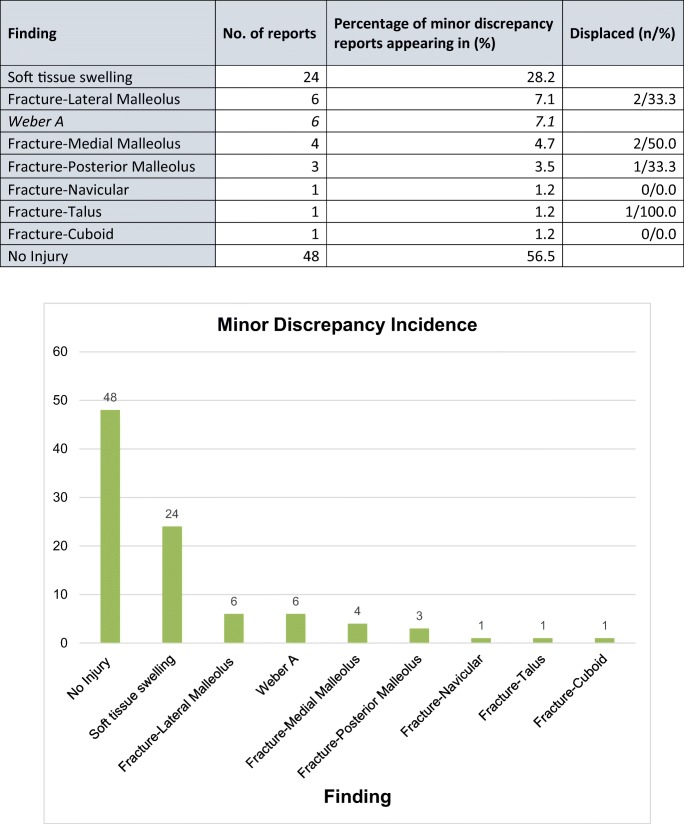
Minor discrepancy incidence

Total incidence of findings across the 85 reports classified as containing minor discrepancy

#### Major discrepancy

The 81 reports with major discrepancy featured 20 (24.7%) with no radiologically observable injury, again representing false positives.

In the remaining 61 reports (75.3%) with missed findings, soft tissue swelling had been initially unreported in 33 (40.7%). There were 22 (27.2%) missed fractures of the lateral malleolus, with 12 (14.8%) categorised as Weber C. A bimalleolar fracture pattern was missed in 2 (2.5%), along with a single missed trimalleolar fracture. Hind foot fractures were missed in a total of 9 (11.1%). There were 5 (6.2%) of unreported navicular fracture, and 1 (1.2%) cuboid fracture See Table 3.

**Table 3:**
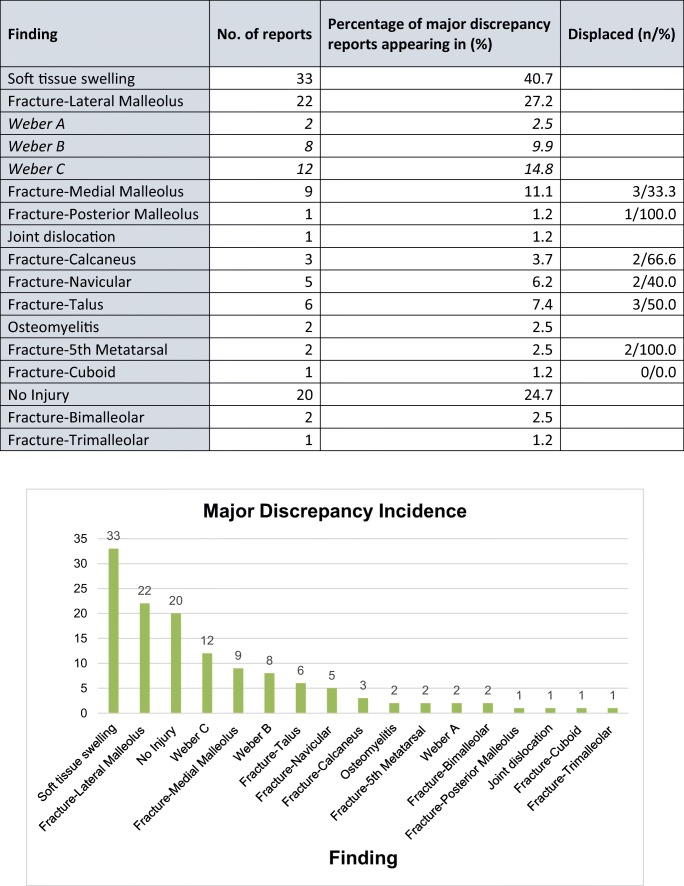
Major discrepancy incidence

Total incidence of findings across the 81 reports classified as containing major discrepancy

#### Missed findings

With regard to unreported findings in bony anatomy, navicular fractures were not initially commented upon in 6 of 15 instances (40.0%); a greatly increased percentage compared to the rate of reporting error across all findings, *p value < 0.0001*. Similarly, 2 of the 6 (33.3%) cuboid fractures were missed, *p value < 0.0001*. Talar (21.9%), Weber A (17.0%), and 5th metatarsal fractures (10.5%) were also disproportionately missed in the initial report.

Only 68 of 1345 initial reports (5.1%) failed to reach the final report’s conclusion of no injury. Fractures of the lateral malleolus were missed in 28 of 484 (5.8%); Weber B fractures being the least commonly missed sub-type with 8 of 315 (2.5%), Weber C with 12 of 122 (9.8%) and Weber A the most with 8 of 47 (17.0%). Only 1 of 104 (1.0%) joint dislocations was initially unreported See Table 4.

**Table 4:**
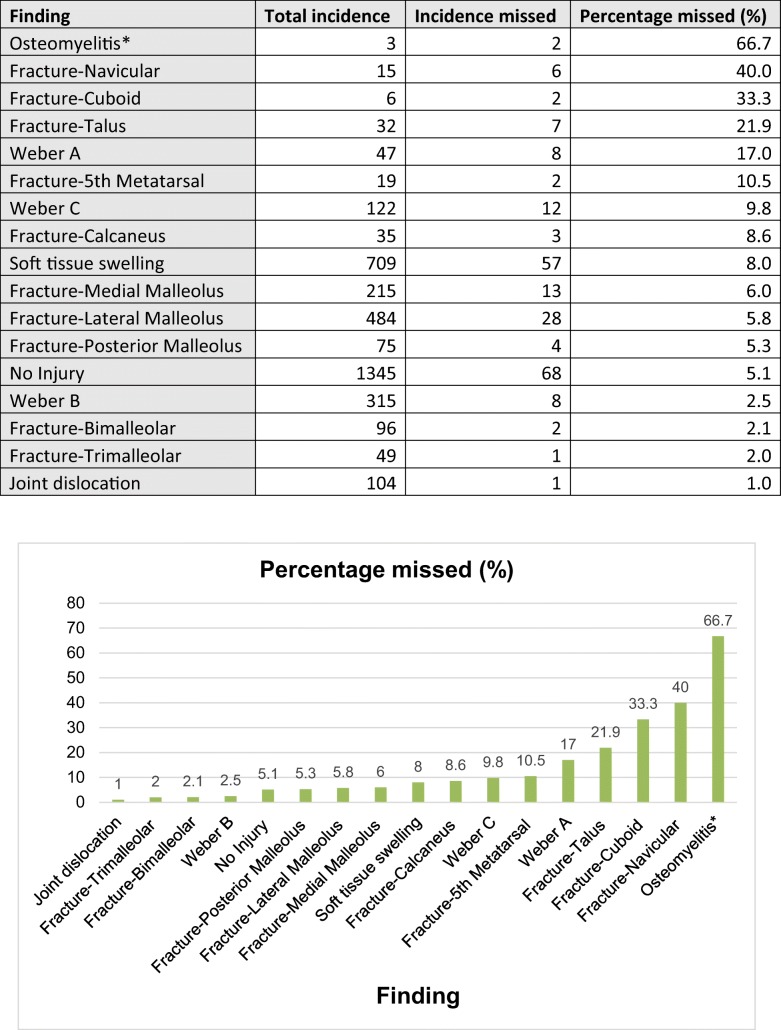
Missed findings

Total incidence of findings against incidence of findings missed on initial report

### Time resolved discrepancy

The time period 08:00–16:59 saw the majority of initial reports being generated; 1503 reports (51.0%) of the total. 472 (16.0%) were generated from 17:00 to 19:59, 530 (18.0%) between 20:00–23-59, and 442 (15.0%) between 00:00–07:59.

During standard working hours, 08:00–16:59, 40 initial reports (49.4%) were generated containing major discrepancy, compared to the 41 expected (51.0%) with a directly proportional relationship to the number of initial reports generated. Those reports with minor discrepancy during this time period related similarly, with 36 observed (42.4%) compared to 43 expected (51.0%).

However, in the time period 00:00–07:59, an increased proportion of reports with major discrepancy were generated; 18 reports (22.2%), compared to an expected 12 (15.0%). Biostatistical analysis for this indicated a Chi Square *p value* = *0.07908*, suggesting this finding narrowly missed the conventional threshold for significance.

In the time period 20:00–23:59 a significant increase in the number of reports with minor discrepancy was observed, 29 (34.1%) compared with 15 expected (18.0%), *p value = 0.00025* See Table 5.

**Table 5:**
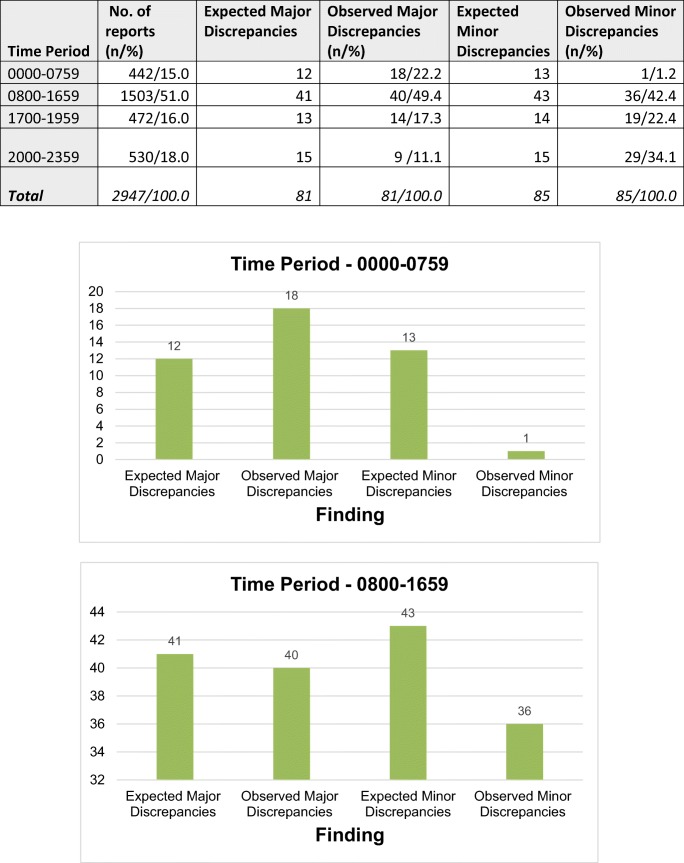

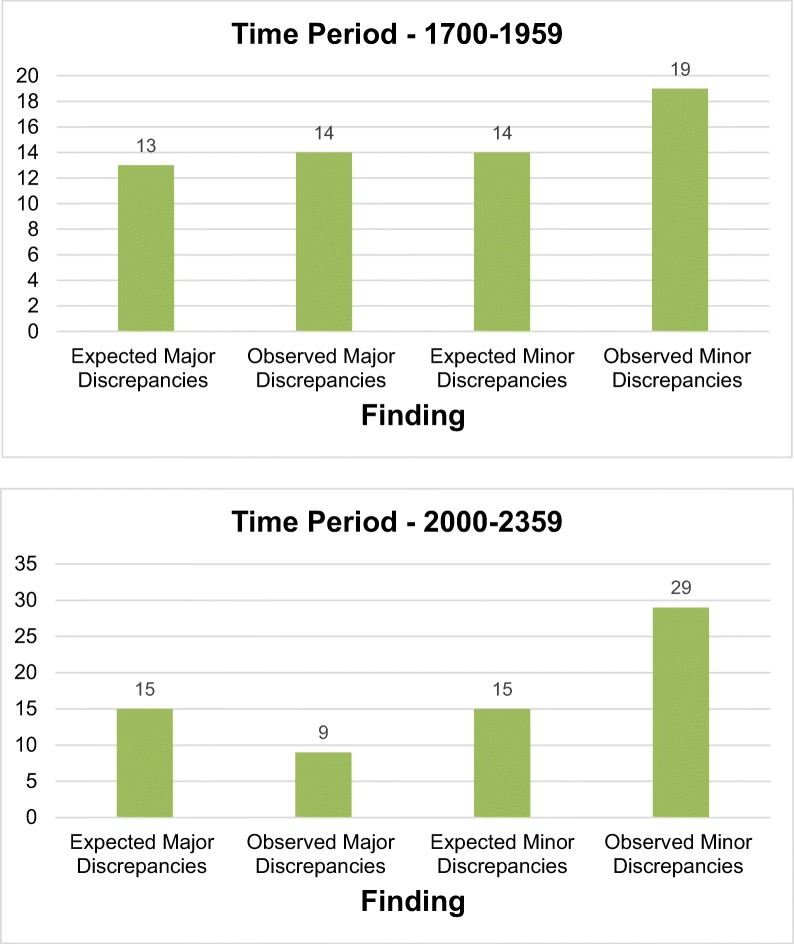
Time resolved discrepancy

## Discussion

### Overall findings

The overall results of this study support earlier findings that fractures of the lateral malleolus represent the most common ankle fractures, with the highest incidence being of Weber type B, followed by type A and then C [10]. There is also strong evidence to support the finding that isolated malleolar injuries make up the majority of ankle fractures, with relatively lower proportions of bimalleolar and trimalleolar fractures [10, 11].

The data does demonstrate somewhat different hindfoot findings than indicated in a review of the existing literature, where fractures of the calcaneus are by far the most common tarsal fractures, seen significantly more frequently than talar fractures [12], (http://www.smo.edu.mx/pdf/0804_publica_talus.pdf), and making up as many as 2% of all fractures [13]. In this study, fractures of both the calcaneus and talus appeared equally in approximately 1% of the included reports. The cause for this is uncertain but in part is likely due to the relatively low numbers of these injuries found within the series.

### Reporting discrepancy

This study demonstrates a reassuringly high accuracy in initial emergency department reporting of ankle X-rays, with discrepancy only being noted in 5.6% of final reports. This compares closely to a 3.8% rate of error seen across all lower limb imaging in a similar study of emergency department interpretation [14]. The reliability of initial X-ray reporting is strong evidence to support its practice in guiding early clinical management, suggesting that radiologically apparent ankle findings can be accurately identified on X-ray by emergency department physicians.

Despite this, the presence of 166 reports containing discrepancy emphasises the need for a timely senior review of ankle X-rays. Of particular note are the 81 cases of major discrepancy where the virtual fracture clinic intervened to make changes to the patient’s management. The majority of these (75.3%) represented a false negative finding, which is likely reflected in the greater potential risk of a missed diagnosis in this setting.

Although appearing relatively infrequently, midfoot fractures were missed with significantly greater regularity than other findings; in total 40.0% of all navicular and 33.3% of all cuboid fractures were missed on the initial report. This is consistent with an existing understanding that their appearance on X-ray can be ‘frequently difficult and occasionally elusive’ [15]. The navicular in particular is of significant importance to the overall stability of the foot and ankle. Its role in forming the keystone of the medial longitudinal arch of the foot means that missed injuries can lead to significant loss of function and pain [16]. Increased awareness of these fractures and their radiological appearance should be promoted in emergency department physicians and those reviewing ankle X-rays.

A total of 3 reports included concerns for osteomyelitis, this was not mentioned in the initial findings of 2 reports. Despite a very high proportion appearing as false negatives, the sensitivity and specificity of X-ray for osteomyelitis is insufficient for accurate diagnosis [17] and as such this finding is of minimal importance.

### Time resolved discrepancy

There has been significant research on hours worked and time of day as potential causes for error in medical practice [9, 18, 19]. This study finds evidence to support the conclusion that ankle X-rays initially reported outside of routine working hours (08:00–16:59) display increased rates of discrepancy. This is true for the time period 00:00–07:59 where an increase in major discrepancies of 7.2% was found, though it should be noted that this narrowly missed the statistical threshold for significance, and from 20:00 to 23:59 where an increase in minor discrepancy of 16.1% was found.

The data used in this study does not precisely reflect the numbers of hours worked by the initial reporting physician and further research is needed to clarify how hours worked may affect this phenomenon independently of time of day. It has also been suggested that higher rates of reporting discrepancy may be attributable to a reduced workload on night shifts [20]. Such an explanation would be consistent with the lower volumes of initial reporting generated outside of 08:00–16:59 and is in-line with the overall trend of the data seen in this study; this makes the hypothesis of reduced workload a compelling explanation for these observations.

### Limitations

A limitation of this study was the high proportion of the preliminary 6886 reports that failed to meet inclusion criteria due to an absent or incomplete assessment of agreement. Although the included 2947 reports represent a significant sample size, the remaining reports would have strengthened the evidence presented. Inertia in the uptake of a new reporting system likely represented the primary reason for this.

Due to the retrospective nature of this research, no inter/intra-reader reliability scoring can be provided between the MSK radiologists and orthopaedic surgeons who made up the virtual fracture clinics. This may leave a degree of unaccounted variability in the final reports, however their consultant level expertise and the routine nature of ankle X-rays serve to mitigate this effect.

### Conclusions

This study found a distribution of ankle X-ray findings consistent with that seen in existing data, with isolated malleolar fractures and specifically Weber type B lateral malleolar fractures representing the most common injuries [10, 11].

Whilst a high standard of accuracy supported the use of initial reporting to determine early clinical management, the presence of 166 reports with discrepancy indicates a continued need for timely senior review of ankle X-rays. Cuboid and navicular fractures were identified as frequently missed and should be a focus of increased awareness amongst initial reporters.

Finally, there was evidence to support an increased rate of errors in initial reporting conducted outside of normal working hours (08:00–16:59). This adds to a growing evidence base suggesting the influence of fatigue, workload, and shift patterns on physician performance.
